# Lentiviral Vectors and Cystic Fibrosis Gene Therapy

**DOI:** 10.3390/v2020395

**Published:** 2010-01-29

**Authors:** Stefano Castellani, Massimo Conese

**Affiliations:** Department of Biomedical Sciences, University of Foggia, Foggia, Italy; E-Mail: s.castellani@unifg.it (S.C.)

**Keywords:** airway epithelium, cystic fibrosis transmembrane conductance regulator (CFTR), lentivirus, lung, *Retroviridae*

## Abstract

Cystic fibrosis (CF) is a chronic autosomic recessive syndrome, caused by mutations in the CF Transmembrane Conductance Regulator (CFTR) gene, a chloride channel expressed on the apical side of the airway epithelial cells. The lack of CFTR activity brings a dysregulated exchange of ions and water through the airway epithelium, one of the main aspects of CF lung disease pathophysiology. Lentiviral (LV) vectors, of the *Retroviridae* family, show interesting properties for CF gene therapy, since they integrate into the host genome and allow long-lasting gene expression. Proof-of-principle that LV vectors can transduce the airway epithelium and correct the basic electrophysiological defect in CF mice has been given. Initial data also demonstrate that LV vectors can be repeatedly administered to the lung and do not give rise to a gross inflammatory process, although they can elicit a T cell-mediated response to the transgene. Future studies will clarify the efficacy and safety profile of LV vectors in new complex animal models with CF, such as ferrets and pigs.

## 1. Introduction

Cystic fibrosis (CF), the most common lethal hereditary disease among Caucasians, with a prevalence of approximately one in 3500 newborns, is characterized by chronic lung infections and inflammation that results in life expectancy being reduced, although treatment advances over the past several decades have raised the median predicted survival age from mid-teens in the 1970s to more than 36 years old today [1].

The CF gene is located on the long arm of chromosome 7 [2] and, based on the prediction of the amino acid sequence of the encoded protein, it was termed CFTR (cystic fibrosis transmembrane conductance regulator). A whole codon deletion, resulting in the loss of a phenylalanine residue at amino acid position 508 (F508del) in the protein is the commonest mutation, accounting for 50–90% of CF patients [3]. The CFTR amino acid sequence contained 12 domains previously recognized as membrane-spanning sequences, and *in vitro* gene transfer experiments demonstrated restoration of chloride channel function in cystic fibrosis pancreatic cells [4]. Further work using purified CFTR protein in phospholipid vesicles confirmed that the protein functions as a cAMP-dependent chloride channel [5].

Lung disease in CF patients reflects chronic infection of the conducting airways with a surprisingly low number of bacterial species. *Staphylococcus aureus* and *Haemophilus influenzae* are early colonizers, whereas *Pseudomonas aeruginosa* and *Burkholderia cepacia* complex (Bcc) often occur later, resulting in progressive loss of lung function and premature death. Several hypotheses link mutations in CFTR to development of lung disease in CF, whose hallmarks are bacterial infection with opportunistic pathogens and a vicious neutrophil-dominated chronic inflammatory response [6–9]. Current available data support the “low volume” hypothesis which postulates that, due to absent chloride transport and increased sodium absorption, the height of the airway surface liquid (ASL) is reduced, leading to impaired mucociliary clearance [10]. Reduced mucociliary clearance leads to formation of thickened dehydrated mucus, which provides an ideal environment for bacterial growth, leading to chronic inflammation and ultimately organ failure in the CF lung. Normal CFTR is partly responsible for maintaining this airway surface liquid volume by means of chloride secretion and down-regulation of the ENaC (epithelial Na+ channel) [11]. *In vitro* models of polarized human cystic fibrosis bronchial epithelial cells demonstrate an isotonic reduction in the volume of airway surface liquid, and consequently impaired movement of the overlying mucus [10]. The importance of CFTR’s regulation of ENaC has been strengthened further by recent data demonstrating that overexpression of ENaC in a mouse model results in many of the key features of cystic fibrosis lung disease (e.g., reduction in airway surface liquid, reduced muco-ciliary clearance and increased mucus plugging of the lungs) without any direct impairment of CFTR function [12].

## 2. Gene therapy of CF lung disease

Cystic fibrosis should be an ideal candidate for gene therapy, for four main reasons: (1) it is a single gene defect; (2) it is a recessive condition, with heterozygotes being phenotypically normal (suggesting gene dosage effects are not critical); (3) the main pathology is in the lung, which is accessible for treatment; and (4) it is a progressive disease with a virtually normal phenotype at birth, offering a therapeutic window.

*In vitro* and *in vivo* studies have suggested that only 5–10% of normal CFTR function is required to reverse the chloride channel defect [13–15], although it is not clear whether this has to be achieved in the majority of the airway epithelial cells, or whether a minority of cells expressing much higher levels would suffice. However, *in vitro* and *in vivo* studies suggest that nearly every cell in the sample must be corrected with CFTR to reverse the excess activity of ENaC [13,16,17]. These findings would imply that gene therapy of CF lung disease should achieve the correction of approximately every cell in the airway epithelium.

In clinical trials to date, two main vector systems have been harnessed to deliver the CFTR cDNA with appropriate promoter into host cells (for reviews, see [18–20]). First, viral vectors with the CFTR cDNA incorporated into the viral genome exploit the efficiency of viruses to enter host cells and achieve relatively high levels of gene expression. Secondly, cationic liposomes complexed with plasmid DNA encoding CFTR enhance the transport of the DNA into host cells. Although cationic liposomes seem to generate a lower immune response than current viral vector systems, the levels of CFTR expression using this delivery system have been relatively poor.

The ideal vector system would have the following characteristics: (1) an adequate carrying capacity; (2) to be undetectable by the immune system; (3) to be non inflammatory; (4) to be safe to the patients with pre-existing lung inflammation; (5) to have an efficiency sufficient to correct the cystic fibrosis phenotype; and (6) to have long duration of expression and/or the ability to be safely re-administered. Furthermore, to provide therapeutic benefit *in vivo*, the delivery of CFTR gene therapy must be extremely efficient to overcome the physical barriers such as thick tenacious mucus and pulmonary surfactant, as well as the logistical difficulties resulting from many viral receptors which are expressed on the basolateral membrane of bronchial epithelial cells. Much of the morbidity and mortality seen in cystic fibrosis patients is related to pre-existing pulmonary inflammation; consequently, it is important that any vector system does not cause further inflammation. Table 1 summarizes the characteristics of viral vectors used in CF gene therapy to date [21–23].

## 3. Lentiviral vectors

Many viral vector systems have been developed for gene therapy applications, but most of these vectors are non-integrating, which limits their usefulness for transgenesis. The best characterized integrating viral vectors originate from the *Retroviridae* family, because of their intrinsic ability to integrate into genomic DNA. However, oncoretroviral vectors have shown limited success because of the requirement of cell replication for integration. Lentiviral vectors (LV) have overcome these limitations showing the ability to transduce nondividing cells and provide a more efficient transgene expression if compared with oncoretroviral vectors. Higher efficiency of LV vectors is likely due to the stability of transcripts derived from the lentiviral provirus because of the strong 3′ poly(A) signal that causes an increased cytoplasmic accumulation of the RNA of the transgene [24]. Naldini *et al*. were the first to demostrate that a LV vector derived from human immunodeficiency virus type 1 (HIV-1) could provide efficient *in vivo* delivery, stable integration and long-term expression of transgenes into non-mitotic cells such as neurons [25,26].

Although various lentiviruses from different species (human, simian, equine, ovine, bovine, feline, caprine) have been used to generate gene transfer vectors (reviewed in [27]) only HIV-1 and feline immunodeficiency virus (FIV) have been considered in the context of airway epithelium and cystic fibrosis gene therapy. Lentiviruses, such as HIV-1, have a diploid genome with two copies of single-stranded plus RNA and a nucleocapsid constituted by the proteins derived from the *gag* gene, viral enzymes such as a protease derived from the *pro* gene, Rnase H, reverse transcriptase and integrase derived from the *pol* gene. The virion is enveloped by the host cell membrane containing the viral protein derived from the *env* gene. After virus entry into the cell, reverse transcription produces viral double strand DNA which enter in the nucleus and integrate randomly and permanently in the host genome, resulting in a provirus state. In the provirus, the lentiviral genome is flanked on both sides by the long terminal repeats (LTR), which contain the U5, R and the U3 regions. The 5′-LTR can act like an RNA pol II promoter. The 3′-LTR acts to terminate transcription and promote polyadenylation. The LTR also has recognition sequences necessary for integration into the genome. Tat is an accessory viral protein which activates the 5′ LTR promoter, whereas Rev controls the amount of RNA splicing as well as the RNA export into the cytoplasm. At the end of cycle infection, viral genomes are packaged, enveloped and released from the cells. Two important *cis*-acting DNA elements, the central polypurine tract sequence (cPPT) and the postregulatory element (PRE), derived from woodchuck hepatitis B virus, are also included in the transfer vector to enhance the transduction efficiency and transcript stability. In particular, cPPT has been shown to facilitate nuclear translocation of pre-integration complexes, and PRE acts at the post-transcriptional level, by promoting nuclear export of transcripts and/or by increasing the efficiency of polyadenylation of the nascent transcript [28–30]. Another important feature in the lentiviral transfer plasmid is a 400-bp deletion in the U3 region of the 3′-LTR, which debilitates the 5′-LTR RNA pol II promoter activity following integration creating a so-called self-inactivating (SIN) vector, increasing the vector biosafety profile [31].

The development of lentiviral vector system, in order to achieve a gene transfer agent, is based on modifications of a native lentivirus genome. Presently, the lentivirus-based gene delivery system is constituted by four components: 1) the packaging elements like structural proteins and enzymes involved in the formation of the viral particles, derived from the *gag-pol* genes; 2) a post-transcriptional regulator for *gag* and *pol* expression as well as nuclear RNA export encoded by the *rev* gene; 3) the vector carrying the transgene to be transferred to the target cells; 4) an heterologous glycoprotein *i.e.*, vesicular stomatitis virus glycoprotein G (VSV-G) which pseudotypes the vector in order to increase its tropism. All structural proteins are HIV-1-derived, except the envelope because of the restricted host range of the HIV-1 env glycoprotein. Association of VSV-G with viral cores derived from lentiviruses, results in an high titer production and in a broad tropism of the vectors. Finally, deletion of the coding sequences for viral gene products renders the LV gene transfer vectors replication incompetent and makes 6–8 kb available for the insertion of desired genes, in this case a large transcriptional units such as an epithelial-specific promoter expressing the CFTR gene [25].

Lentivirus vectors only recently made their debut in human clinical trials for hematological disorders, such as β-thalassemia and sickle cell disease [32]. In the first reported clinical trial with LV vectors, an HIV-1 derived vector expressing an antisense gene against the HIV-1 envelope gene (termed VRX496) was transferred to autologous CD4 T cells. A single intravenous infusion of these gene-modified autologous T cells was well tolerated in all five patients enrolled in the clinical trial; self-limiting mobilization of the vector and improvement of immune function was observed in four out of five patients [33]. Currently there are 21 clinical trials with LV vectors referring to various diseases, representing 1.4% of the vectors used in gene therapy clinical trials, as reported by http://www.wiley.co.uk/genetherapy/clinical/.

## 4. Barriers to efficient viral-mediated gene transfer to the airway epithelium

Gene therapy of cystic fibrosis lung disease needs an efficient delivery and a persistent expression of the CFTR transgene into the airway epithelium. LV vectors appear to be promising vehicles for gene delivery into respiratory epithelial cells due to their ability to infect nondividing cells and to integrate into the host cell genome mediating long-term persistence of transgene expression. Various physical and pathological barriers, inherent also to CF lung, including mucus plugs, glycocalyx components and mucins at the apical surface of the epithelia, represent an obstacle for viral attachment to the target cells and inhibit viral transduction from the luminal face [34,35]. These hurdles, together with immune response to vector encoded proteins [36], limit the success of *in vivo* application of gene transfer vectors in the airway epithelium. Moreover, the expression of receptors for many commonly used viral vectors is more abundant on the basolateral membrane than on the apical side of the respiratory epithelium and they are hardly accessible because of the airway tight junctions [37–39].

## 5. Gene tranfer to the airway epithelium mediated by LV vectors

To mimic the persistent and progressive infection typical for CF patients, the bacteria can be encapsulated into agar beads and injected to the mouse lung via the trachea [40]. The impact of chronic *Pseudomonas aeruginosa* infection on adenovirus-mediated gene transfer was evaluated by two studies [41,42], which demonstrated that efficiency of adenovirus-mediated gene transfer to the airway epithelium is significantly reduced by inflammation induced by *Pseudomonas.* Increased adenovirus-specific CD8 cytotoxic T cell activity was considered by the authors as one of the underlying mechanisms [42]. Recently, we have demonstrated that LV VSV-G vector-mediated transduction of the airway epithelium is not affected when administered to the lung of chronically infected mice [43]. Although these data refer to only one day post-injection, our findings together with those previously published underscore the importance of considering the influence of the disease milieu when evaluating modes of gene therapy for such diseases in animal models.

Other studies have shown that the capacity of LV vectors to transduce a fully differentiated respiratory epithelium is lower compared with an undifferentiated one. A first generation VSV-G pseudotyped HIV vector was able to transduce wild type CFTR into poorly differentiated human bronchial xenografts and its expression corrected the chloride transport defect and reverted the bacterial killing activity; however gene transfer was less efficient when the epithelium was fully differentiated [44]. A second generation LV vector could enable efficient and long-term transduction of a 3D spheroid culture of fetal human airway epithelial cells derived from the fetal trachea or from fetal airway xenografts (a culture model of airway epithelial cells in suspension showing a polarized, junctional and differentiated mucociliary surface epithelium) for up to 80 days [45]. LV vector-mediated LacZ expression in the human fetal tracheal xenograft model was observed in up to 99% of the surface epithelial cells and the submucosal gland cells, up to nine months after vector administration [46], indicating that HIV-1-derived vectors mediate long-term gene expression by targeting a ‘progenitor cell’ compartment.

**Figure 1.**
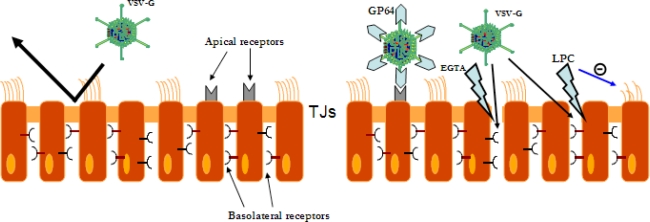
VSV-G LV vectors have been shown to be inefficient in *in vivo* gene transfer into a fully differentiated epithelium unless treatment with agents that disrupt the TJ integrity (EGTA; LPC) exposing basolateral receptors to the vector. LPC acts also increasing vector residence time by reducing cilial beat (blue arrow). To overcome the inability of VSV-G LV to efficiently infect the airway epithelium in the absence of injuring agents other heterologous envelope glycoproteins than VSV-G (e.g., GP64 from baculovirus *A. Californica*) conferred to the LV vector the ability to transduce differentiated airway epithelium from the apical surface.

Pseudotyping the vectors with heterologous envelopes and pre-conditioning of the airway tight junctions are the strategies currently used to overcome the paucity of receptors for lentivirus on the apical surface of the respiratory epithelium and to allow the access to the basolateral compartement receptors (Figure 1).

Most of the studies on HIV-1-derived lentiviral mediated gene transfer in the airway epithelium refer to gene delivery mediated by LV vectors pseudotyped with VSV-G which is useful not only to allow virus particle concentration, but also to modulate virus interaction with the host immune system and to broaden its host range.

VSV-G LV vectors have been shown to be inefficient in *in vivo* gene transfer into a fully differentiated epithelium unless disruption of the epithelial barrier integrity obtained with calcium-chelating agents like EGTA [47], inhalation exposure to sulfur dioxide [48], or modification of the barrier function of the airway epithelium with lysophosphatidylcholine (LPC) [49] is obtained. As a proof-of-principle that disruption of tight junction integrity helps LV-mediated gene transfer to a differentiated airway epithelium, Johnson *et al*. demostrated that direct *in vivo* delivery of VSV-G pseudotyped HIV-1- based vector to the nasal epithelium of rats and mice failed to mediate gene transfer, but injury of the epithelium with sulfur dioxide (SO_2_) 1–2 hours before vector delivery strongly enhanced transduction levels of the nasal epithelium. SO_2_ pre-treatment also enhanced gene transfer to the tracheas of rodents [48].

Calcium-chelating agents may be more useful in the context of CF lung disease, since they transiently disrupt tight junctions [50]. CFTR function was restored after *in vitro* transduction of fully differentiated human CF airway epithelia with a VSV-G pseudotyped FIV vector formulated with EGTA [47]. In the same study pre-treatment of rabbit tracheas with EGTA solution for 30–60 minutes prior to vector application resulted in transduction mostly of lower airways (basal cells, Clara cells, and alveolar type II cells). Gene transfer persisted in respiratory epithelial cells for 6 weeks.

The attachment and entry mechanism of VSV-G pseudotyped LV vectors into the airway epithelial cells and subsequent infection are still unknown. We demonstrated a role of glycosaminoglycans (GAGs) in gene transfer mediated by a third generation VSV-G pseudotyped LV vector in an *in vitro* model of polarized airway epithelial cells. In particular, heparan sulfate, chondroitin sulfate A and B seem to be strongly involved in the infection of respiratory cells mediated by a VSV-G pseudotyped LV vector [51]. However, one hour of treatment with 12 mM EGTA significantly increased VSV-G LV efficiency transduction in polarized cells obtained from a CF F508del homozygous patient [51], indicating that not all the putative attachment receptors are expressed on the apical surface of CF airway epithelium.

Other biological modifers of the membrane permeability have been actively searched [22]. LPC pre-treatment results in a transient disruption of tight junctions, facilitating the access of lentiviral vectors to the basolateral membrane [49]. Other mechanisms by which LPC might enhance gene transfer are by acting as a mucolytic agent and by reducing cilial beat, both of which increase vector residence time [52]. Pre-treatment of the epithelium with 1% LPC 1 hour prior to intranasal instillation of a VSV-G-LV vector expressing LacZ reporter gene in mice produced significant transgene expression in the nasal respiratory epithelium for at least 92 days, suggesting that transduction of airway progenitor cells had occurred. Transduction with LV expressing CFTR after LPC treatment achieved sustained CFTR expression and partial correction of the electrophysiological defect in the nose of CF mice for at least 110 days [49].

In a recent work, a VSV-G pseudotyped LV carrying LacZ or CFTR gene was delivered in the nostrils of mice 1 hour after pre-treatment with 0,3 % LPC via inhalation-driven instillation. LacZ transgene expression lasted over 24 months and was restricted to the anterior regions of transitional and respiratory epithelium within the dosed nasal airway affecting primarily ciliated and nonciliated cells, but also a low number of secretory and basal cells. At 24 months when LacZ gene expression was still present, the number of cells transduced was significantly reduced compared to the initial 1 week level. Significant correction of CFTR function, measuring nasal potential, was present at 1 and 12 months compared to untreated mice [53]. Although these findings are consistent with an approximate 3-month cell turnover time originally established for murine tracheal airway [54], recent data suggest longer turnover times (up to 17 months) in deeper regions of the mouse lung [55].Thus, it is currently unclear, if prolonged expression is due to vector integration into the pulmonary stem or progenitor cells or due to the long life-expectancy of airway epithelial cells.

Agents disrupting tight junction structure and disturbing membrane permeability may not be useful in the context of CF lung disease, since CF lungs are replenished with bacterial products and inflammatory mediators. To overcome the inability of VSV-G-pseudotyped lentiviral vectors to efficiently infect the airway epithelium in the absence of injuring agents, other heterologous envelope glycoproteins, such as those from the Marburg virus or the Zaire subtype Ebola virus [56,57], the baculovirus *Autografa californica* [58] and the influenza virus [59], were used to pseudotype the vectors.

HIV-1-based vectors pseudotyped with the envelope from the Zaire strain of the Ebola virus (EboZ) gave efficient transduction (70% of positive cells) when applied to the apical side of polarized epithelia [56]. *Ex-vivo* transduction of human trachea by EboZ-pseudotyped vector resulted in high levels of LacZ expression as compared to VSV-G pseudotyped vector. When the Ebo-Z pseudotyped virus was injected in the trachea of mice, 30% of the entire tracheal epithelium was transduced at day 28 and 24% at day 63. Interestingly, high expression was observed in submucosal glands, while transduction efficiency was lower in epithelia of more distal lung (airway and alveolar) cells.

Pseudotyping with the GP64 derived from the baculovirus *A. californica* conferred to a FIV lentiviral vector the ability to transduce differentiated human airway epithelia from the apical surface with higher efficiency than VSV-G LV [58]. Intranasal instillation of the vector carrying LacZ formulated in viscous methylcellulose carrier, which is thought to act by increasing vector residence time, resulted in persistent gene expression in the mouse nose, with gene expression observed one year post-infection.

Kremer *et al*. compared the efficiency of transduction of mice nasal airways by a VSV-G pseudotyped vector (carrying LacZ gene) after a pre-treatment with LPC, and the same vector pseudotyped with GP64 envelope without any pre-treatment. In the absence of pre-conditioning the GP64-pseudotyped lentiviral vector resulted significantly less efficient than the VSV-G pseudotyped vector after LPC treatment. The levels of transduction were similar for the two vectors when a pre-treatment with LPC was used. The cell types transduced were essentially the same with the majority of cells transduced being respiratory (ciliated cells). However, while the VSV-G LV resulted in persisting gene expression, transduction with the GP64 LV resulted in gene expression that decline to undetectable levels over six months, whether or not an LPC treatment was used. These results suggest that the GP64-pseudotyped LV vector, in contrast to the VSV-G-pseudotyped vector, was unable to transduce progenitor cells for respiratory epithelium, even when used in combination with LPC pre-treatment [52].

Different results in transducing airway epithelial cells *in vitro* and *in vivo* were obtained with other envelope proteins, such as those from influenza virus [59], Ross River virus [60], and Jaagsiekte sheep retrovirus [61]. For example, McKay *et al*. demostrated that an HIV-1-derived LV vector pseudotyped with influenza virus hemagglutinin could efficiently transduce, via the apical membrane, differentiated cultures of human and murine airway epithelial cells, and mouse tracheal epithelium *in vivo*, with a preference for ciliated cells [59]. These vectors warrant further studies about their attachment and entry mechanisms.

## 6. Safety of LV vectors in the lung

Gene therapy viral vectors are known to elicit transgene- or viral protein-specific immune responses [62]. This issue has long been studied with adenoviral vectors, with which, transgene expression *in vivo* usually extinguishes within 2 to 3 weeks, concurrent with the development of inflammation. This is caused by the rapid activation of potent CD8 and CD4 T cell responses against both the viral antigens and the transgene. In addition, activation of B cells by viral capsid proteins, leading to the production of neutralizing antibodies, limits effective readministration of the vector. When administered to the lung, adenoviral vectors activate cytolytic transgene-specific T cells [63]. This drawback has hindered the development of adenovirus as a gene therapy vector for CF. A preliminary evaluation of safety of administration of LV vectors into airways showed that *in vivo* delivery of a HIV-1-derived vector in the tracheas of C57Bl/6 mice did not induce inflammatory infiltrates post-infection [56], and the persistent expression in mice and rabbits suggested a potential “immune tolerance” in these animal models [47]. Intravenous administration of LV vectors in immunocompetent mice induced the proinflammatory cell-adhesion molecules ICAM-1, VCAM-1, PECAM-1 and the costimulatory molecule B7-2 [64] and resulted in reduced duration of expression of the transgene [65], whereas direct administration into the retina, liver, muscle, and brain has been associated with milder systemic inflammatory and immune responses [66–68].

A GP64-pseudotyped FIV vector was also used to investigate the efficacy of repeated administration to the airways [69]. In particular the authors reported for the first time a successful *in vivo* re-administration of lentiviral vectors to respiratory epithelia, associated with an increase expression of reporter or therapeutic transgenes, without the development of innate and adaptive responses that represent a significant limitation to clinical applications for airway diseases for other virus vectors, such as adenoviruses or adenoassociated viruses. In this study, GP64-FIV was repeatedly delivered to murine nasal epithelia, in particular four groups of mice received 1, 3, 5 or 7 doses of the vector carrying the luciferase transgene over the same number of consecutive days. Mice underwent bioluminescence imaging 1, 4, 8 and 12 weeks following the final dose demonstrating additive increases in transgene expression with repeat dosing, probably due both to an increase of percentage of cells expressing a transgene and in the number of transgene copies/cells. The levels of anti-GP64 IgG were measured in sera and in bronchoalveolar lavage (BAL) fluid one week after a booster dose of the vector, showing low levels of anti-GP64 IgG antibodies in sera for all groups of mice and higher levels in BAL fluid; importantly, the levels of inactivating antibodies measured with a neutralizing antibody assay resulted below the limit of detection indicating that though an adaptive immune response is mounted against the vector, it is insufficient to block gene transfer. Although these results are encouraging for the expression of a therapeutic protein over the life, recently published data argue against this scenario. Limberis and colleagues demonstrated that transient expression of Green Fluorescent Protein (GFP) at day 90 in alveolar epithelium following an intratracheal injection of VSV-G-pseudotyped HIV-1-derived vector to the mouse lung is due to transgene- and *gag*-specific T-cell activation [70]. These results suggest that issues linked to the elicitation of an immune response are also associated with the application of pseudotyped LV vectors for gene therapy of pulmonary genetic diseases, such as CF. Specifically, it has been predicted, using computational analysis, the likelihood of a CFTR-specific T-cell activation following expression of the therapeutic CFTR gene in CF subjects with the most common mutation F508del [71]. It is thus likely that expression of the therapeutic CFTR in CF patients with the F508del mutation could be subject to antigen cross-presentation or activation of quiescent CFTR-specific T cells, an issue that has been understimated in previous clinical studies. Indeed, Limberis and colleagues identified a dominant CD8 T cells epitope in the CFTR gene upon intramuscular injection of an adenovirus vector expressing human wild-type CFTR cDNA into CFTR KO mice [72].

Lineage-regulated transgene expression will be necessary for effective, long-term gene therapies for disorders affecting the lung. Most gene therapy clinical trials directed to respiratory epithelium to date have used constitutive promoters such as the promoter from the cytomegalovirus (CMV) or the promoter in the retroviral LTR. However, long-term animal studies have indicated that, over time, transgene expression can be silenced from viral promoters [73,74], or can disregulate cellular genes near the integration site leading to tumorigenesis [75]. Thus, lineage-specific regulation of expression will be beneficial for many gene therapy applications to avoid vector silencing or toxicities associated with unregulated transgene expression. Furthermore, lung-specific transgene expression would have the advantage of avoiding transgene expression in antigen presenting cells, such as dendritic cells, and limiting immune responses against the transgene and viral encoded genes.

Hendrickson *et al*. constructed a series of lentiviral vectors with regulatory elements to obtain lung-specific transgene expression: the surfactant protein C promoter (SPC) for alveolar epithelial type II cell (AECII) expression, the Clara cell 10-kDa protein (CC10) for Clara cell expression in the airway, and the Jaagsiekte sheep retrovirus (JSRV) promoter for expression in both cell types. Transgene expression from the SPC and CC10 vectors was restricted to alveolar epithelial type II and Clara cell lines, respectively, while expression from the JSRV vector was observed in multiple respiratory and non-respiratory cell types. After intratracheal delivery of lentiviral vector to mice, transgene expression was observed in AECII from the SPC lentiviral vector, and in Clara cells from the CC10-promoted lentivector. Transgene expression was not detected in non-respiratory tissues after intravenous delivery of CC10 and SPC lentiviral vectors [76].

Innate immune responses to LV vectors in the context of lung gene therapy have not been thoroughly investigated. Epithelial respiratory cells function as part of the innate immune responses and not merely only as a physical barrier. Multiple substances with pro- and anti-inflammatory as well as antimicrobial activities are secreted by airway epithelial cells [77–79]. Airway epithelial cells can sense and are activated by respiratory pathogens, including viruses [80,81]. On this line, we have undertaken an evaluation of the pro-inflammatory potential of LV vectors *in vitro* in airway epithelial cells. A third-generation VSV-G LV vector did not induce those pro-inflammatory signals usually observed in human respiratory epithelial cells exposed to classical stimuli, such as TNF-α, or other gene transfer vectors previously studied, such as adenovirus-derived vectors, except for a mild and transient induction of transcription of IFN-γ in an epithelial tracheal cell line [82]. These results, although indicating “stealth” properties of LV vectors in respect with pro-inflammatory potential, warrants further evaluation in preclinical animal models. Confirming our results in those models, there would be nevertheless the undoubted necessity to cope with adaptive immune response, as shown by Limberis and colleagues.

## 7. LV-mediated gene transfer into fetal airway epithelium

Recent studies have explored the feasibility of LV vector-mediated gene expression in the fetal airway epithelium, considering the advantage of a therapeutic intervention before clinical onset and in the absence of a functional immune response. *In vivo* GFP gene transfer to fetal respiratory epithelium has been achieved in rabbits after direct VSV-G LV intra-tracheal or intra-amniotic administration, hovewer tracheal delivery was associated with high fetal mortality rate (75%) and an elevated degree of transgene expression in the control ipsilateral uninjected fetuses, likely due to contamination of fetomaternal circulation, compared with intra-amniotic administration [83].

Injection of VSV-G LV carrying GFP reporter gene into the lung parenchima of fetuses of rhesus monkeys resulted in transgene expression in pulmonary vasculature and alveolar cells in term fetuses and three month aged pups [84]. This work also indicates that postnatal lung development and function were not altered after fetal intraorgan gene transfer and subsequent transgene expression prenatally and postnatally.

In a recent study an HIV-based lentiviral pseudotyped vector with the baculovirus GP64 envelope was applied to the fetal, neonatal or adult airways. Fetal intra-amniotic administration resulted in transduction of approximately 14% of airway epithelial cells, including both ciliated and non-ciliated epithelia of the upper, mid and lower airways, with a negligible alveolar or nasal transduction. A significant transduction of the airway epithelium mainly in distal lung and alveoli was detected at 1 year after application following neonatal intra-nasal administration, while in the adult the majority of transduction was restricted to alveoli. In contrast, VSV-G pseudotyped virus transduced only after adult and neonatal application and no transduction was observed after fetal administration [85].

Taken together, these results strongly indicate that fetal airway epithelium can be transduced by LV vectors and that the pseudotype can be fundamental as in the transduction of adult airway epithelium. The early intervention with an integrating gene transfer vector able to transduce the lung via the lumen, avoiding immune elimination of transgenic protein and probably increasing lung stem-cell transduction to result in a persistent gene expression, while ethically complex, may be a successful therapeutic approach for CF.

## 8. Conclusions and perspectives

LV vectors are endowed with interesting features which make them promising agents for application to gene therapy of CF lung disease. However, they present some drawbacks which need to be addressed before they can enter into the clinic. Although the issue of attachment and entry mechanisms has not been studied in detail, it seems that, at least in *in vitro* cultures of human polarized airway epithelia and small conventional animal models, the apical surface of the airway epithelium poses a strenous barrier to efficient transduction from this side, due to the lack of apical receptors. Moreover, while other viral vectors have been considered in light of the extracellular barriers, such as the thick and dehydrathed mucus layer covering the airway epithelium, comprehensive studies on this aspect are still lacking. The alternative to the use of tight junction disrupting agents and membrane detergents, which could possibly enhance the leakage of bacterial products and inflammatory mediators into the submucosa and further damage the CF lung, is the pseudotyping of LV envelope with heterologous proteins from other viruses. This approach should be evaluated in terms of the safety issue, when pathogen viruses are used [57]. Another approach is to use physical methods in order to enhance the residence time of viral vectors onto the airway epithelium, avoiding their wash out by the mucociliary clearance. For example, magnetofection is a nucleic acid delivery technique to cells supported and site-specifically guided by the attractive forces of magnetic fields acting on viral vectors which are associated with magnetic nanoparticles. This field is actively investigated by us and other research groups [86,87].

Most of the data on the efficacy (*i.e.*, the correction of the electrophysiological defect) of LV vectors have been collected in CF mouse models. For both anatomical and physiological reasons, mice do not show a respiratory syndrome that is equivalent to that found in humans [88]. To develop meaningful LV vectors for CF gene therapy, evaluation of LV vectors should be done on novel animal models for CF. Pigs and ferrets seem to be better animal models to develop protocols and vectors for gene therapy of CF, because they share many anatomical and physiologycal similarities with those of human lungs [89,90].

The issue of immune response to transgene- and viral genes is a caveat in the use of viral vectors for human gene therapy. The first indications on the elicitation of innate and adaptive responses to LV vectors have been produced, although this field is in its infancy. It seems that LV vectors have a low pro-inflammatory potential, while they can stimulate a transgene-directed T cell response. Novel animal models may aid also in elucidating this issue.

## Figures and Tables

**Table 1.** Comparison among viral vectors for CF gene therapy.

**Vector**	**Packaging Capacity**	**Integration**		**Persistence of Expression**	**Pro-inflammatory/Immunogenicity**	**Biosafety and efficiency studies on animal models**	**Efficacy studies on animal models (with CFTR transgene)**	**Clinical Trials for CF**
**Adenovirus**	8 kb		No	No	High	Yes	Yes	Yes
**Adeno-associated virus**	5 kb		Both episomal and integrated gene expression	Yes	Low	Yes	Yes	Yes
**Lentivirus**	8 kb		Yes	Yes	Low	Yes	Yes	No
